# Cardiovascular-kidney-metabolic Syndrome: a current and urgent concept

**DOI:** 10.1590/2175-8239-JBN-2024-0277en

**Published:** 2025-05-19

**Authors:** Andrea Pio de Abreu, Luciano Ferreira Drager, Madson Queiroz Almeida, Luiz Aparecido Bortolotto, Heno Ferreira Lopes

**Affiliations:** 1 Universidade de São Paulo, Faculdade de Medicina, Hospital das Clínicas, Departamento de Nefrologia, São Paulo, SP, Brazil.; 2 Universidade de São Paulo, Faculdade de Medicina, Hospital das Clínicas, Instituto do Coração, Unidade de Hipertensão São Paulo, SP, Brazil.; 3 Universidade de São Paulo, Faculdade de Medicina, Hospital das Clínicas, Departamento de Endocrinologia e Metabologia, São Paulo, SP, Brazil.

**Keywords:** Cardiovascular Diseases, Renal Insufficiency, Chronic, Obesity, Chronic Inflammation, Oxidative Stress

## Abstract

The concept of cardiovascular-kidney-metabolic health is based on the idea of a multifaceted interaction among several cardiovascular, renal, and meta­bolic factors. Alterations in this complex interaction have a significant impact on morbidity and mortality. The ramifications of poor cardiovascular-kidney-metabolic health are far-reaching, with a notable clinical impact. A considerable proportion of the population is affected by precarious cardiovascular, renal, and metabolic health, and indivi­duals with adverse social determinants of health endure an even greater burden. To this end, it is essential to develop an approach that incorporates metabolic staging and prioritizes lifelong prevention. Equally imperative is integrating the social determinants of health into care models for Cardiovascular-kidney-metabolic syndrome, facilitating patient-centered interdisciplinary care. The American Heart Association (AHA) provides guidelines on the definition, staging, paradigms, and holistic approaches to the management of patients with this syndrome. Furthermore, it offers a comprehensive framework for effectively and equitably improving the cardiovascular, kidney, and metabolic health of the population.

## Introduction

Cardiovascular-Kidney-Metabolic Syn­drome (CKM) represents a complex inter­connection between the cardiovascular, renal, and metabolic systems, constituting a new paradigm in the understanding and treatment of interconnected diseases^1^. This concept emerged from the need to integrate therapeutic approaches for clinical conditions that frequently coexist and share common pathophysiological mechanisms, such as insulin resistance, chronic inflammation, and endothelial dysfunction^2^. The high prevalence and significant impact of these conditions on public health underscore the importance of an integrated approach to their management^3^.

CKM Syndrome is particularly relevant due to its association with highly prevalent chronic diseases such as type 2 diabetes mellitus (T2DM), obesity, cardiovascular disease (CVD), and chronic kidney disease (CKD)^4^. Epidemiological studies indicate that the coexistence of these conditions leads to an exponential increase in morbidity and mortality, primarily due to cardiovascular events^5^. In addition, the overlap of metabolic and cardiovascular risk factors underscores the need for an integrated approach to the diagnosis, treatment, and prevention of these diseases^6^.

The pathophysiological mechanisms linking the cardiovascular, renal, and metabolic systems are complex and multifaceted^7^. Insulin resistance and chronic inflammation, commonly associated with T2DM and hypertension, play a crucial role in the progression of CKM Syndrome promoting endothelial alterations that ultimately lead to vascular and renal dysfunction^8^. These processes are exacerbated by oxidative stress and the activation of the renin-angiotensin-aldosterone system (RAAS). Both play a key role in the pathogenesis of these interconnected diseases^9^.

Effective management of CKM Syndrome requires therapeutic strategies that simultaneously address the multiple risk factors involved^10^. This includes strict glycemic control, blood pressure management, and obesity treatment through lifestyle interventions, pharmacological therapies, and, when indicated, metabolic surgery^11^. Evidence suggests that early and integrated actions may prevent CKM Syndrome progression, in addition to significantly improving patients’ quality of life and clinical outcomes^12^.

Finally, early screening and prevention are essential pillars in addressing CKM Syndrome^13^. The implementation of screening programs that identify high-risk individuals at an early stage could reduce the incidence of serious complications and promote more effective interventions^14^. In this context, interdisciplinary collaboration among nephrologists, cardiologists, and endocrinologists, along with a multidisciplinary team, is crucial for designing personalized and integrated treatment plans that consider the complex interactions between the cardiovascular, renal, and metabolic systems^1^.

## A New Approach: The Interdependence of The Cardiovascular, Renal, and Metabolic Systems

The CKM Syndrome emerged from the observation of the interdependence between the cardiovascular, renal, and metabolic systems. Although knowledge of this interdependence is long-standing, the concept/nomenclature proposed and suggested by the American Heart Association (AHA) is novel. The new nomenclature aims to inform the medical and multi-professional communities of the need to incorporate CKD into the former concept of metabolic syndrome and CVD, recognizing the significant global impact of CKD on morbidity and mortality of CKM Syndrome patients. Originally described by Ndumele et al.^1^, the CKM Syndrome proposes that dysfunctions in these systems do not occur in isolation but rather as part of a pathological *continuum*. This concept emerged from the need for an integrative approach, as patients with cardiovascular disease often also experience renal and metabolic complications. Thus, CKM Syndrome represents an evolution in the understanding and treatment of these conditions, highlighting the complexity and interconnection between these systems.

The mechanisms underlying CKM Syndrome include chronic inflammation, oxidative stress, and endothelial dysfunction^5^. The interrelationship between hypertension, T2DM, and CKD illustrates how dysregulation in one system could precipitate or exacerbate dysfunction in others^2^. For instance, insulin resistance is associated with hypertension, which can in turn result in renal damage, causing or worsening CKD. This vicious circle is sustained by the activation of the renin-angiotensin-aldosterone system (RAAS) and the production of inflammatory mediators, all of which together promote multisystem dysfunction^7^.

Recent studies have shown that CKM Syndrome is associated with a significant increase in morbidity and mortality^3^. In a recent review article, Cases et al.^3^ suggest that early and integrated interventions could lead to improved clinical outcomes. Clinical evidence indicates that adopting a comprehensive approach, which considers interactions between the cardiovascular, renal, and metabolic systems, may reduce adverse events and improve patients’ quality of life. Data from epidemiological studies also reveal that individuals with multiple conditions associated with CKM Syndrome have a worse prognosis than those with a single condition^4^.

Furthermore, the adoption of the CKM Syndrome concept in clinical practice may favor more integrated and effective approaches in the management of patients with multiple comorbidities^10^. Identifying and treating CKM Syndrome comprehensively can reduce disease progression and improve patients’ quality of life. Integrating the medical specialties involved contributes to the successful implementation of this approach. For example, integrated manage­ment among cardiologists, nephrologists, and endocrinologists could lead to better care coordination and more effective interventions^1,12,13,14,15^.

### What we Need to Know About CKM Syndrome?

Excessive or dysfunctional adiposity is thought to be the substrate of CKM Syndrome. Obesity, especially visceral obesity, is associated with chronic inflammation and insulin resistance, key factors in the pathogenesis of CKM Syndrome^1,2^. Improving integrated care for obesity is essential to prevent the progression of this syndrome. Interventions aimed at weight loss, lifestyle modification, and management of metabolic risk factors are crucial to delaying or preventing the development of cardiovascular and renal complications^3^. Furthermore, obesity plays a central role in endothelial dysfunction and RAAS activation, contributing to the progression of CKM Syndrome^4^.

The risk of CKM Syndrome starts in the early stages of life and is exacerbated over time by adverse social determinants of health (SDH)^1,5^. Factors such as low income, lack of access to quality healthcare, and limited education are critical determinants that influence the prevalence and severity of CKM Syndrome. Public health interventions addressing these social determinants are essential to reduce the burden of CKM Syndrome and promote health equity. Promoting a healthy lifestyle from childhood, including a balanced diet and regular physical activity, could help mitigate the risks associated with CKM Syndrome^6^.

Periodic determination of albuminuria and renal function are key elements in predicting CVD in CKD patients, both in early and advanced stages^7^. Albuminuria is an early biomarker of kidney and cardiovascular damage, while glomerular filtration rate (GFR) is an important indicator of renal function. Monitoring these parameters allows for the early detection of complications and the implementation of appropriate interventions to prevent disease progression. Studies indicate that intensive blood pressure control and proper glycemic control are key to protecting renal function and reducing cardiovascular risk in CKD patients^8^.

Novel drugs and treatments for the management of CKM Syndrome should be prescribed comprehensively and equitably, with an emphasis on implementation in populations at greater risk of disease progression^1^. Drugs such as sodium-glucose cotransporter 2 (SGLT2) inhibitors, glucagon-like peptide-1 (GLP-1) receptor agonists, finerenone, and recent potassium binders have demonstrated significant benefits in reducing cardiovascular events and providing renal protection^10^. The introduction of these new medications has attracted growing interest from various medical specialties in addition to nephrologists, including cardiologists, endocrinologists, general practitioners, geriatricians, and others.

Accessibility to these treatments aims to ensure that all patients, regardless of their socioeconomic status, may benefit from therapeutic advances in the management of CKM Syndrome.

CKM Syndrome, which originates from excess dysfunctional adipose tissue, involves the interaction of some chronic noncommunicable diseases (NCDs), all of which are priorities due to their impact on morbidity and mortality. NCDs refer to long-lasting, slow-process pathologies, such as CVD, T2DM, and CKD, which often result from a combination of modifiable and non-modifiable risk factors, including genetic, metabolic, environmental, and behavioral factors. The recognition of CKM Syndrome as a global public health priority is crucial, precisely because it integrates several NCDs, reinforcing the need for integrated strategies in the management of chronic diseases^11^. Therefore, implementing health policies that promote prevention, early diagnosis, and integrated treatment could significantly contribute to improving clinical outcomes and reducing the global burden of these interconnected conditions.

## American Heart Association (AHA) Presidential Guideline on Cardiovascular-Kidney-Metabolic Health

CKM Syndrome is defined as a health disorder resulting from the connections between obesity, diabetes, CKD, and CVD (heart failure, atrial fibrillation, coronary heart disease, stroke, and peripheral artery disease). This syndrome includes both individuals at risk of developing CVD and those with established CVD.

**Figure 1:**
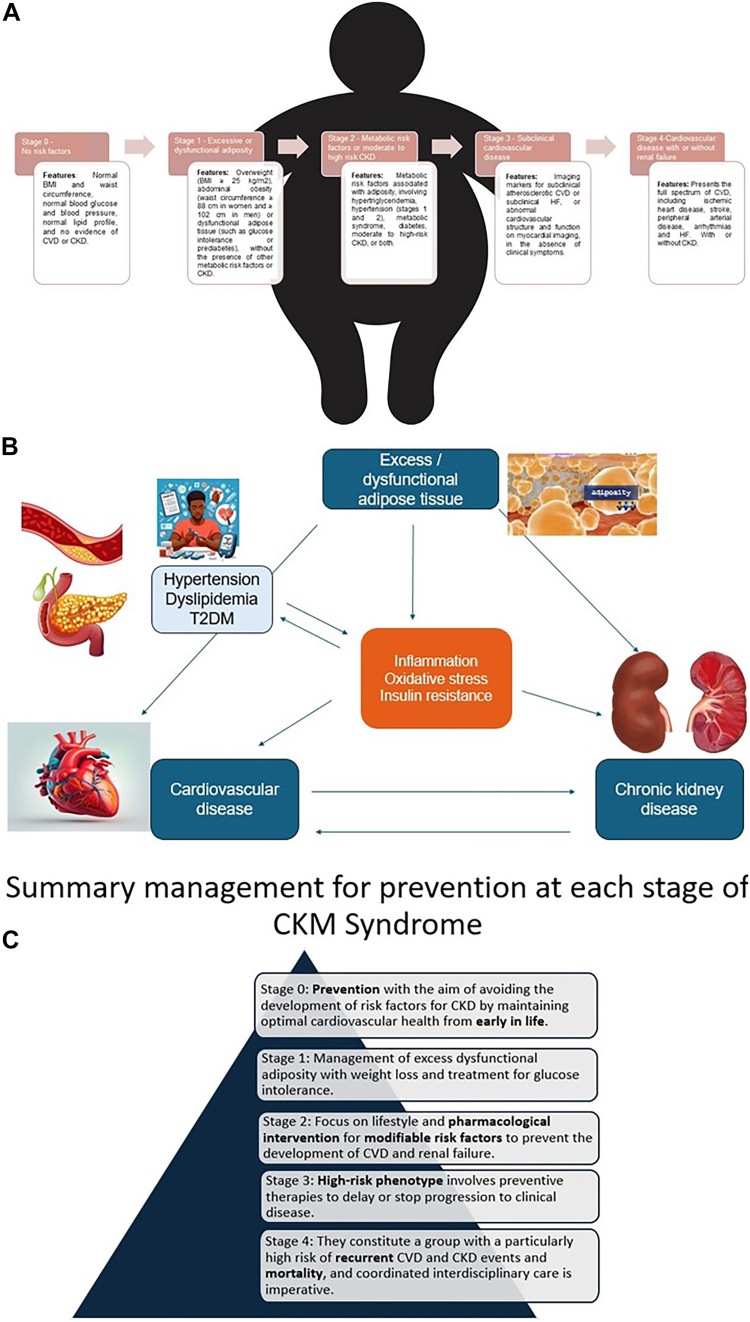
(A) Different stages of Cardiovascular-kidney-metabolic syndrome (CKM) and main characteristics of each stage; (B) Vicious circle of CKM Syndrome. (C) Summary management for prevention at each stage of CKM Syndrome.

The guideline provides a staging framework for CKM Syndrome that reflects its pathophysiology, risk spectrum, and opportunities for prevention and care optimization. The stages are classified as follows: stage 0 (no risk factors), stage 1 (excessive or dysfunctional adiposity), stage 2 (metabolic risk factors or moderate to high-risk CKD), stage 3 (subclinical CVD or CKM Syndrome risk equivalents), and stage 4 (clinical CVD in CKM)^1^. In addition, risk-enhancing factors influence progression through the stages of CKM Syndrome (see Figure 1).

Abbreviations – CHD: coronary heart disease; CKD: chronic kidney disease; CVD: cardiovascular disease; HF: heart failure; BMI: body mass index.

Screening for CKM Syndrome risk factors is recommended throughout life to improve prevention and management approaches in both young people and adults. The frequency of screening should be adjusted according to the CKM Syndrome stage. In the early stages, when no evident risk factors are present, a more spaced-out screening schedule may be adopted, focusing on annual assessments of basic indicators such as body mass index (BMI), blood pressure, and lipid profile. As risk factors begin to emerge, such as excessive or dysfunctional adiposity, hypertension, and insulin resistance, screening should become more frequent, with biannual or quarterly monitoring, including detailed blood tests and kidney function assessments. In this context, it is important to emphasize that the CKM Syndrome staging distinguishes between elevated BMI and dysfunctional adiposity, by inserting the latter concept into stage 1 of the syndrome. We should indeed consider that central obesity is at the very core of the problem.

Excessive or dysfunctional adiposity should be addressed through lifestyle modifications, with an emphasis on weight loss, to prevent progression and facilitate regression across CKM Syndrome stages^7^. The adoption of healthy eating habits, increased physical activity, and behavioral interventions are essential in this process. Supervised weight loss programs and continuous support from healthcare professionals could significantly contribute to reducing the risk associated with obesity. Studies show that a modest weight loss - greater than 5% of initial body weight – may lead to significant improvements in cardiovascular risk factors^16^.

The choice of cardioprotective hypoglycemic agents in the treatment of patients with diabetes may optimize cardiovascular risk reduction^7^. SGLT2 inhibitors are prioritized for patients with chronic kidney disease (CKD), heart failure, or high risk of heart failure, due to their proven benefits in reducing cardiovascular morbidity and mortality^17^. Conversely, GLP-1 receptor agonists are especially recommended for patients with uncontrolled hyperglycemia, patients on high doses of insulin, or those who are severely obese, due to their significant effect on weight loss and glycemic control^17^. The combination of both drugs may be considered in patients with multiple risk factors within the CKM Syndrome, offering a robust and comprehensive therapeutic approach. Although the AHA’s position does not specifically focus on metabolic surgery, it is necessary to emphasize that this is also an effective therapeutic possibility, with a significant impact on reducing renal and cardiovascular outcomes, being considered a safe procedure even for CKD patients^18^. In more advanced stages, where clinical manifestations of CKM Syndrome, such as subclinical or established cardiovascular disease, are already present, the AHA guideline recommends even more rigorous screening, incorporating advanced diagnostic methods such as echocardiography, stress tests, and continuous monitoring of critical parameters, aimed at early and effective intervention.

**Figure 2:**
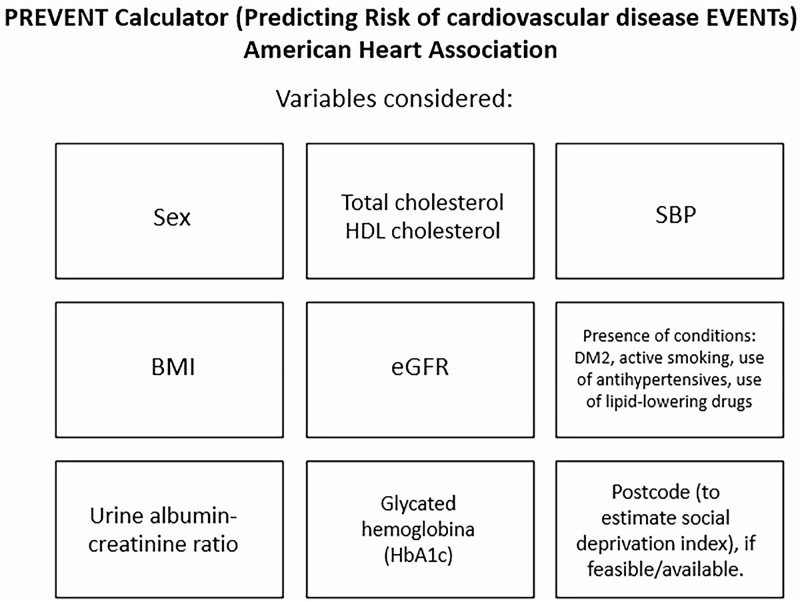
Variables included in the PREVENT risk calculator.

New approaches to predict outcomes related to CKM Syndrome have been described. Among these, a new cardiovascular risk score called “PREVENT” (Predicting Risk of CVD Event)^19^, duly validated by the AHA^20^. The proposal of this new score, certainly one of the highlights of the current guideline, expands on the former AHA “ASCVD Risk”, allowing 10 and 30-year risk estimates for events such as myocardial infarction, stroke, and heart failure (atherosclerotic cardiovascular disease). Intended for primary prevention patients, the tool provides 10-year risk estimates for individuals aged 30 to 79, as well as up to 30-year risk estimates for those aged 30 to 59. The variables considered in the PREVENT score are shown in Figure 2.

Abbreviations – HDL: high-density lipoprotein; SBP: systolic blood pressure; eGFR: estimated glomerular filtration rate; BMI: body mass index.

Another important approach of this guideline relates to SDH. Given the excessive burden of CKM Syndrome among individuals with SDH and the significant impact of these determinants on the management and outcomes of the syndrome, systematic screening for individuals with SDH is emphasized. Incorporating these determinants into risk prediction and clinical care for patients with the syndrome could substantially improve clinical outcomes. Screening should include identifying factors such as poverty, limited access to healthcare, inadequate education, and unfavorable working conditions, which may exacerbate the progression of CKM Syndrome. Thus, addressing these factors is essential for more effective and equitable care, promoting a broader perspective in the management of patients’ health.

### What’s New in the Nephrology Scenario?

In the context of nephrology, the AHA position provides an important recommendation: clinicians should assess the urinary albumin-to-creatinine ratio, in addition to the estimated GFR, in patients with CKD, T2DM, hypertension, and metabolic syndrome, to fully characterize the risk of CKD and CVD, particularly heart failure. These low-cost measures are crucial for the early detection of complications and continuous monitoring of kidney disease progression. Guidelines for the proper use of nephroprotective therapies with cardiovascular benefits, such as the traditional and well-established angiotensin-converting enzyme inhibitors (ACEIs), angiotensin II receptor blockers (ARBs), and SGLT2 inhibitors, are provided with the aim of ensuring effective and integrated management of these conditions.

### What are the Challenges in Implementing CKM Syndrome?

Despite the advancements in understanding CKM Syndrome, there are still challenges in implementing the concept of the syndrome in clinical practice^6^. The heterogeneity of clinical presentations and the need for personalized treatment strategies represent significant barriers. Furthermore, the lack of clear guidelines for the integrated management of CKM Syndrome and the need for greater interdisciplinary training among healthcare professionals represent additional obstacles. Individualizing treatment, considering the specificities of each patient, is essential for overcoming these challenges and optimizing clinical outcomes^9^.

Continuous research is needed to clarify the mechanisms underlying CKM Syndrome and to develop specific therapeutic interventions. Longitudinal studies and randomized clinical trials are crucial to validating integrated treatment approaches. In addition, the development of new technologies and biomarkers may facilitate early detection and monitoring of the syndrome. Interdisciplinary collaboration and the integration of new scientific findings into clinical practice are key to making progress in the management of this complex syndrome.

A comprehensive framework for optimizing CKM Syndrome health in the population should also include improving health education, investing in research focused on the syndrome, and the systematic evaluation of SDH. Improved obesity management and the availability of integrated teams to support lifestyle changes and weight control are essential components of this approach. It is necessary to recognize obesity as a chronic disease that requires a comprehensive, stigma-free approach. In addition, increasing equitable access to pharmacotherapies, supporting interdisciplinary care models, applying proven implementation strategies in healthcare centers, and developing partnerships to support optimal cardiovascular health in diverse communities are critical to the successful CKM Syndrome management. These initiatives not only contribute to improving clinical outcomes but also foster a more equitable and sustainable approach to the health of all affected patients.

## Conclusion and Final Message

CKM Syndrome offers a new perspective on the interrelationship among cardiovascular, renal, and metabolic diseases. Integrating therapeutic approaches for these conditions, which share common pathophysiological mechanisms such as insulin resistance, chronic inflammation, and endothelial dysfunction, is essential for improving patient management and clinical outcomes. The adoption of prevention strategies, early screening, and integrated treatment could significantly reduce the morbidity and mortality associated with these interconnected diseases. Furthermore, interdisciplinary collaboration among cardiologists, nephrologists, endocrinologists, and other healthcare professionals is crucial for developing personalized and integrated treatment plans that consider the complex interactions between the cardiovascular, renal, and metabolic systems. It is worth noting that the new CKM Syndrome concept “embraces” the significance of chronic kidney disease. While the classic concept of metabolic syndrome represents a set of metabolic alterations that affect cardiovascular risk, CKM Syndrome offers a new perspective, in which metabolic alterations resulting from dysfunctional obesity are linked to cardiac and renal alterations, configuring a cycle of dysfunction that requires a global approach. In the current context, where the world is experiencing a pandemic of obesity, T2DM, hypertension, and CKD, CKM Syndrome has emerged as a current and extremely important concept.
