# Supplementary material for: Cardiovascular-kidney-metabolic Syndrome: a current and urgent concept
Source: J Bras Nefrol. 2025 May 19;47(2):e20240277. doi: 10.1590/2175-8239-JBN-2024-0277en (PMC12092022; doi:10.1590/2175-8239-JBN-2024-0277en)
Supplement: Translation

# Translation: Síndrome cardiovascular-renal-metabólica: um conceito atual e urgente

**DOI:** 10.1590/2175-8239-JBN-2024-0277pt

**Authors:** Andrea Pio de Abreu, Luciano Ferreira Drager, Madson Queiroz Almeida, Luiz Aparecido Bortolotto, Heno Ferreira Lopes

**Affiliations:** 1 Universidade de São Paulo, Faculdade de Medicina, Hospital das Clínicas, Departamento de Nefrologia, São Paulo, SP, Brasil.; 2 Universidade de São Paulo, Faculdade de Medicina, Hospital das Clínicas, Instituto do Coração, Unidade de Hipertensão São Paulo, SP, Brasil.; 3 Universidade de São Paulo, Faculdade de Medicina, Hospital das Clínicas, Departamento de Endocrinologia e Metabologia, São Paulo, SP, Brasil.

**Keywords:** Doenças Cardiovasculares, Insuficiência Renal Crônica, Obesidade, Inflamação Crônica, Estresse Oxidativo

## Abstract

O conceito de saúde cardiovascular-renal-metabólica baseia-se na noção de uma interação multifacetada entre diversos fatores cardiovasculares, renais e metabólicos. Alterações nesta interação complexa exercem um impacto significativo na morbidade e na mortalidade. As ramificações de uma saúde cardiovascular, renal e metabólica deficientes são de grande alcance, com um impacto clínico notável. Uma proporção considerável da população é afetada por uma saúde cardiovascular, renal e metabólica precárias, e as pessoas com determinantes sociais de saúde adversos suportam um ônus ainda maior. Para isso, é essencial desenvolver uma abordagem que incorpore o estadiamento metabólico e priorize a prevenção ao longo da vida. É igualmente imperativo integrar os determinantes sociais da saúde aos modelos de atendimento à síndrome cardiovascular-renal-metabólica, facilitando os cuidados interdisciplinares centrados no paciente. A *American Heart Association* (AHA) oferece diretrizes sobre a definição, o estadiamento, os paradigmas e as abordagens holísticas para o manejo de pacientes com tal síndrome. Além disso, oferece uma visão abrangente para melhorar de forma eficaz e equitativa a saúde cardiovascular, renal e metabólica da população.

## Introdução

A Síndrome Cardiovascular-Renal-Metabólica (SCRM) representa uma interconexão complexa entre os sistemas cardiovascular, renal e metabólico, constituindo um novo paradigma na compreensão e no tratamento de doenças interconectadas^1^. Esse conceito surgiu da necessidade de integrar as abordagens terapêuticas para condições clínicas que frequentemente coexistem e compartilham mecanismos fisiopatológicos comuns, como resistência à insulina, inflamação crônica e disfunção endotelial^2^. A elevada prevalência e o impacto significativo dessas condições sobre a saúde pública ressaltam a importância de uma visão integrada para o seu manejo^3^.

A SCRM é particularmente relevante devido à sua associação com doenças crônicas altamente prevalentes, como diabetes mellitus tipo 2 (DM2), obesidade, doenças cardiovasculares (DCV) e doença renal crônica (DRC)^4^. Estudos epidemiológicos indicam que a coexistência dessas condições resulta em um aumento exponencial na morbidade e mortalidade, principalmente devido a eventos cardiovasculares^5^. Além disso, a sobreposição de fatores de risco metabólicos e cardiovasculares reforça a necessidade de uma abordagem integrada para o diagnóstico, tratamento e prevenção dessas doenças^6^.

Os mecanismos fisiopatológicos que ligam os sistemas cardiovascular, renal e metabólico são complexos e multifacetados^7^. A resistência à insulina e a inflamação crônica, comumente associadas ao DM2 e à hipertensão arterial, desempenham um papel fundamental na progressão da SCRM, promovendo alterações endoteliais que culminam em disfunção vascular e renal^8^. Esses processos são exacerbados pelo estresse oxidativo e pela ativação do sistema renina-angiotensina-aldosterona (RAAS), ambos cruciais na patogênese dessas doenças interligadas^9^.

A condução eficaz da SCRM requer estratégias terapêuticas que abordem, de forma simultânea, os múltiplos fatores de risco envolvidos^10^. Isso inclui o controle glicêmico rigoroso, o manejo da pressão arterial e o tratamento da obesidade por meio de intervenções no estilo de vida, intervenções farmacológicas e, quando indicado, cirurgia metabólica^11^. Evidências sugerem que ações precoces e integradas podem prevenir a progressão da SCRM, além de melhorar significativamente a qualidade de vida dos pacientes e os desfechos clínicos^12^.

Por fim, a triagem precoce e a prevenção são pilares essenciais na abordagem da SCRM^13^. A implementação de programas de triagem que identifiquem precocemente indivíduos de maior risco pode reduzir a incidência de complicações graves e promover intervenções mais eficazes^14^. Neste contexto, a colaboração interdisciplinar entre nefrologistas, cardiologistas e endocrinologistas, juntamente com uma equipe multidisciplinar, é fundamental para a elaboração de planos de tratamento personalizados e integrados, que considerem as complexas interações entre os sistemas cardiovascular, renal e metabólico^1^.

## Uma Nova Abordagem: A Interdependência dos Sistemas Cardiovascular, Renal E Metabólico

A SCRM surgiu a partir da observação da interdependência entre os sistemas cardiovascular, renal e metabólico. Embora o conhecimento dessa interdependência seja antigo, o conceito/nomenclatura proposto e sugerido pela *American Heart Association* (AHA) é novo. A nova nomenclatura tem como objetivo divulgar para a comunidade médica e multiprofissional a necessidade de incorporar a DRC ao antigo conceito da síndrome metabólica e da DCV, reconhecendo o impacto global significativo da DRC na morbi-mortalidade de pacientes portadores de SCRM. Originalmente descrita por Ndumele et al.^1^, a SCRM propõe que as disfunções nesses sistemas não ocorram isoladamente, e sim como parte de um *continuum* patológico. Esse conceito emergiu da necessidade de uma abordagem integrativa, uma vez que pacientes com doenças cardiovasculares frequentemente também apresentam complicações renais e metabólicas. Dessa forma, a SCRM representa uma evolução na forma de entender e tratar essas condições, evidenciando a complexidade e a interconexão entre esses sistemas.

Os mecanismos subjacentes à SCRM incluem inflamação crônica, estresse oxidativo e disfunção endotelial^5^. A inter-relação entre hipertensão, DM2 e DRC ilustra como a desregulação em um sistema pode precipitar ou agravar disfunções em outros^2^. Por exemplo, a resistência à insulina associa-se à hipertensão, que, por sua vez, pode danificar os rins, provocando ou agravando a DRC. Este círculo vicioso é sustentado pela ativação do sistema renina-angiotensina-aldosterona (SRAA) e pela produção de mediadores inflamatórios, os quais, em conjunto, promovem uma disfunção multissistêmica^7^.

Estudos recentes demonstram que a SCRM está associada a um aumento significativo na morbidade e mortalidade^3^. Em um artigo de revisão recente, Cases et al.^3^ sugerem que intervenções precoces e integradas podem levar à melhoria dos resultados clínicos. Evidências clínicas indicam que a adoção de uma abordagem ampla, que considere as interações entre os sistemas cardiovascular, renal e metabólico, pode reduzir eventos adversos e melhorar a qualidade de vida dos pacientes. Dados de estudos epidemiológicos também revelam que indivíduos com múltiplas condições associadas à SCRM apresentam um prognóstico pior do que aqueles com uma única condição^4^.

Além disso, a adoção do conceito de SCRM na prática clínica pode favorecer abordagens mais integradas e eficazes no manejo de pacientes com múltiplas comorbidades^10^. Identificar e tratar a SCRM de forma abrangente pode reduzir a progressão de doenças e melhorar a qualidade de vida dos pacientes. A integração das especialidades médicas envolvidas contribui para a implementação bem-sucedida desta abordagem. Por exemplo, o manejo integrado entre cardiologistas, nefrologistas e endocrinologistas pode levar a uma melhor coordenação do cuidado e a intervenções mais eficazes^1,12–15^.

### O Que Precisamos Saber Sobre a Scrm?

Supõe-se que a adiposidade excessiva ou disfuncional seja o substrato da SCRM. A obesidade, especialmente a obesidade visceral, está associada à inflamação crônica e à resistência à insulina, fatores-chave na patogênese da SCRM^1,2^. Melhorar o cuidado integrado à obesidade é essencial para prevenir a progressão desta síndrome. Intervenções que visam à perda de peso, à modificação do estilo de vida e ao manejo dos fatores de risco metabólicos são cruciais para retardar ou impedir o desenvolvimento de complicações cardiovasculares e renais^3^. Além disso, a obesidade desempenha um papel central na disfunção endotelial e na ativação do SRAA, contribuindo para a progressão da SCRM^4^.

O risco da SCRM começa já nas primeiras fases da vida e se agrava pelos determinantes sociais adversos da saúde (DSAS) ao longo do tempo^1,5^. Fatores como baixa renda, falta de acesso a cuidados de saúde de qualidade e educação limitada são determinantes críticos que influenciam a prevalência e a gravidade da SCRM. Intervenções de saúde pública que abordem esses determinantes sociais são essenciais para reduzir a carga da SCRM e promover a equidade na saúde. A promoção de um estilo de vida saudável desde a infância, incluindo uma dieta equilibrada e a prática regular de atividade física, pode ajudar a mitigar os riscos associados à SCRM^6^.

A determinação periódica da albuminúria e da função renal são elementos centrais para prever DCV em pacientes com DRC, tanto em estágios iniciais quanto avançados^7^. A albuminúria é um marcador precoce de dano renal e cardiovascular, enquanto a taxa de filtração glomerular (TFG) é um importante indicador da função renal. O monitoramento desses parâmetros permite a detecção precoce de complicações e a implementação de intervenções adequadas para prevenir a progressão da doença. Estudos indicam que o controle intensivo da pressão arterial e o controle glicêmico adequado são fundamentais para proteger a função renal e reduzir o risco cardiovascular em pacientes com DRC^8^.

Novos medicamentos e tratamentos destinados ao manejo da SCRM devem ser prescritos de forma ampla e equitativa, com ênfase na implementação em populações com maior risco de progressão da doença^1^. Medicamentos como os inibidores do cotransportador sódio-glicose 2 (SGLT2), os agonistas do receptor do peptídeo-1 semelhante ao glucagon (GLP-1), finerenona e recentes quelantes de potássio têm demonstrado benefícios significativos na redução de eventos cardiovasculares e na proteção renal^10^. A introdução desses novos medicamentos tem despertado crescente interesse por parte de diversas especialidades médicas além dos nefrologistas, incluindo cardiologistas, endocrinologistas, clínicos gerais e geriatras, dentre outros.

A acessibilidade a esses tratamentos visa assegurar que todos os pacientes, independentemente de sua condição socioeconômica, possam se beneficiar dos avanços terapêuticos no manejo da SCRM.

A SCRM, cuja origem está associada ao excesso de tecido adiposo disfuncional, envolve a interação de algumas doenças crônicas não transmissíveis (DCNT), todas prioritárias devido ao seu impacto na morbimortalidade. As DCNT referem-se a patologias de longa duração e processos lentos, como DCV, DM2 e DRC, que frequentemente resultam da combinação de fatores de risco modificáveis e não modificáveis, como genéticos, metabólicos, ambientais e comportamentais. O reconhecimento da SCRM como uma prioridade de saúde pública global é fundamental, justamente por integrar diversas DCNT, reforçando a necessidade de estratégias integradas para o manejo de doenças crônicas^11^. Portanto, a implementação de políticas de saúde que promovam a prevenção, o diagnóstico precoce e o tratamento integrado pode contribuir significativamente para a melhora dos desfechos clínicos e a redução da carga global dessas condições interligadas.

## Diretriz Presidencial da *American Heart Association* (AHA) Sobre Saúde Cardiovascular-Renal-Metabólica

A SCRM é definida como um distúrbio de saúde decorrente das conexões entre obesidade, diabetes, DRC e DCV (insuficiência cardíaca, fibrilação atrial, doença coronariana, acidente vascular cerebral e doença arterial periférica). Essa síndrome inclui tanto indivíduos em risco de desenvolver DCV quanto aqueles com DCV já estabelecida.

A diretriz estabelece uma estrutura de estadiamento da SCRM que reflete a fisiopatologia, o espectro de risco e as oportunidades de prevenção e otimização do cuidado envolvendo a síndrome. Os estágios são classificados da seguinte forma: estágio 0 (ausência de fatores de risco), estágio 1 (adiposidade excessiva ou disfuncional), estágio 2 (fatores de risco metabólicos ou DRC de risco moderado a alto), estágio 3 (DCV subclínica ou equivalentes de risco na SCRM), e estágio 4 (DCV clínica na SCRM)^1^. Além disso, fatores que aumentam o risco influenciam a progressão ao longo dos estágios da SCRM (ver Figura 1).

**Figura 1:** (A) Diferentes estágios da Síndrome Cardiovascular-Renal-Metabólica (SCRM) e características principais de cada estágio; (B) Círculo vicioso da SCRM. (C) Manejo resumido para prevenção em cada estágio da SCRM.

Abreviações – DC: doença cardíaca; DRC: doença renal crônica; DCV: doença cardiovascular; IC: insuficiência cardíaca; IMC: índice de massa corpórea.

A triagem para fatores de risco da SCRM é recomendada ao longo de toda a vida, com o objetivo de aprimorar as abordagens de prevenção e manejo em jovens e adultos. A frequência da triagem deve ser ajustada de acordo com o estágio da SCRM. Nos estágios iniciais, quando não há fatores de risco evidentes, pode-se adotar uma periodicidade mais espaçada, focando em avaliações anuais de indicadores básicos como o índice de massa corporal (IMC), pressão arterial e perfil lipídico. À medida que os fatores de risco começam a surgir, como adiposidade excessiva ou disfuncional, hipertensão arterial e resistência à insulina, a triagem deve se tornar mais frequente, com monitoramentos semestrais ou trimestrais, incluindo exames de sangue detalhados e avaliações da função renal. Nesse contexto, é importante ressaltar que o estadiamento da SCRM considera a diferença entre o IMC elevado e a adiposidade disfuncional, ao inserir este último conceito no estágio 1 da síndrome. Devemos considerar, de fato, que a obesidade central é o próprio cerne do problema.

A adiposidade excessiva ou disfuncional deve ser abordada por meio de modificações no estilo de vida, com ênfase na perda de peso, a fim de prevenir a progressão e facilitar a regressão ao longo dos estágios da SCRM^7^. A adoção de hábitos alimentares saudáveis, aumento da atividade física e intervenções comportamentais são fundamentais nesse processo. Programas de perda de peso supervisionados e o suporte contínuo de profissionais de saúde podem contribuir significativamente para a redução do risco associado à obesidade. Estudos mostram que uma perda de peso modesta, superior a 5% do peso corporal inicial, pode levar a melhorias significativas nos fatores de risco cardiovasculares^16^.

A escolha de agentes hipoglicemiantes cardio­protetores no tratamento de pacientes com diabetes pode otimizar a redução do risco cardiovascular^7^. Os inibidores de SGLT2 são priorizados para pacientes com doença renal crônica (DRC), insuficiência cardíaca ou alto risco de insuficiência cardíaca, devido aos seus benefícios comprovados na redução da morbidade e mortalidade cardiovascular^17^. Por outro lado, os agonistas do receptor GLP-1 são especialmente recomendados para pacientes com hiperglicemia não controlada, pacientes em uso de altas doses de insulina ou obesos graves, por seu efeito significativo na perda de peso e controle glicêmico^17^. A combinação de ambos os medicamentos pode ser considerada em pacientes com múltiplos fatores de risco dentro da SCRM, oferecendo uma abordagem terapêutica robusta e abrangente. Embora o posicionamento da AHA não foque especificamente na cirurgia metabólica, faz-se necessário destacar que também se trata de uma possibilidade terapêutica efetiva, com impacto significativo na redução de desfechos renais e cardiovasculares, sendo considerado um procedimento seguro, inclusive, para pacientes com DRC^18^. Em estágios mais avançados, onde já existem manifestações clínicas da SCRM, como doença cardiovascular subclínica ou estabelecida, a diretriz da AHA recomenda uma triagem ainda mais rigorosa, incorporando métodos diagnósticos avançados como o ecocardiograma, testes de esforço e monitoramento contínuo de parâmetros críticos, visando uma intervenção precoce e eficaz.

Novas abordagens para prever os desfechos relacionados à SCRM foram descritas. Dentre estas, um novo escore de risco cardiovascular denominado “PREVENT” (*Predicting Risk of CVD Event*)^19^, devidamente validado pela AHA^20^. A proposta deste novo escore, certamente um dos pontos altos da presente diretriz, expande o antigo “*ASCVD Risk*” da AHA, permitindo estimativas de risco de 10 e 30 anos para eventos como infarto, AVC e insuficiência cardíaca (doença cardiovascular aterosclerótica). Destinada a pacientes de prevenção primária, a ferramenta fornece estimativas de risco de 10 anos para indivíduos de 30 a 79 anos, assim como estimativas de risco de até 30 anos para aqueles entre 30 e 59 anos. As variáveis consideradas no escore PREVENT encontram-se na Figura 2.

**Figura 2:** Variáveis compreendidas na calculadora de risco PREVENT.

Abreviações – HDL: lipoproteína de alta densidade; PAS: pressão arterial sistólica; TFGe: Taxa de Filtração glomerular estimada; IMC: índice de massa corporal.

Outra abordagem importante da presente diretriz está relacionada às DSAS. Dada a carga excessiva da SCRM entre indivíduos com DSAS e o impacto significativo desses determinantes no manejo e nos resultados da síndrome, destaca-se a triagem sistemática para indivíduos com DSAS. Incorporar esses determinantes na previsão de risco e no atendimento clínico para pacientes com a síndrome pode melhorar substancialmente os desfechos clínicos. A triagem deve incluir a identificação de fatores como pobreza, acesso limitado a cuidados de saúde, educação inadequada e condições de trabalho desfavoráveis, que podem exacerbar a progressão da SCRM. Desse modo, abordar esses fatores é essencial para um cuidado mais eficaz e equitativo, promovendo uma visão mais ampla na gestão da saúde dos pacientes.

### O que há de Novo no Cenário Nefrológico?

No contexto da nefrologia, o posicionamento da AHA traz uma importante recomendação: os clínicos devem avaliar a razão albumina-creatinina urinária, além da TFG estimada, em pacientes com DRC, DM2, hipertensão e síndrome metabólica, a fim de caracterizar completamente o risco de DRC e DCV, particularmente insuficiência cardíaca. Essas medidas, de baixo custo, são cruciais para a detecção precoce de complicações e para o monitoramento contínuo da progressão da doença renal. As orientações para o uso adequado de terapias nefroprotetoras com benefícios cardiovasculares, como os tradicionais e consolidados inibidores da enzima conversora de angiotensina (IECA), bloqueadores do receptor de angiotensina II (BRA) e inibidores de SGLT2, são fornecidos com o objetivo de garantir um manejo eficaz e integrado dessas condições.

### Quais os Desafios para a Implementação da SCRM?

Apesar dos avanços na compreensão da SCRM, ainda há desafios na implementação do conceito da síndrome na prática clínica^6^. A heterogeneidade das apresentações clínicas e a necessidade de estratégias de tratamento personalizadas representam barreiras significativas. Além disso, a falta de diretrizes claras para o manejo integrado da SCRM e a necessidade de maior capacitação interdisciplinar entre os profissionais de saúde constituem obstáculos adicionais. A individualização do tratamento, levando em consideração as especificidades de cada paciente, é essencial para superar esses desafios e otimizar os resultados clínicos^9^.

A pesquisa contínua é necessária para esclarecer os mecanismos subjacentes à SCRM e desenvolver intervenções terapêuticas específicas. Estudos longitudinais e ensaios clínicos randomizados são cruciais para validar abordagens de tratamento integradas. Além disso, o desenvolvimento de novas tecnologias e biomarcadores pode facilitar a detecção precoce e o monitoramento da síndrome. A colaboração interdisciplinar e a integração de novas descobertas científicas na prática clínica são fundamentais para avançar no manejo dessa síndrome complexa.

Uma estrutura abrangente para otimizar a saúde da SCRM na população deve incluir também a melhoria da educação em saúde, o investimento em pesquisas voltadas à síndrome e a avaliação sistemática dos DSAS. A melhoria do manejo da obesidade e a disponibilidade de equipes integradas para apoiar mudanças no estilo de vida e controle do peso são componentes essenciais dessa abordagem. Faz-se necessário reconhecer a obesidade como uma doença crônica, que necessita de uma abordagem ampla e livre de estigmas. Além disso, aumentar o acesso equitativo a farmacoterapias, apoiar modelos de cuidado interdisciplinar, aplicar estratégias comprovadas de implementação em centros de saúde e desenvolver parcerias para apoiar a saúde cardiovascular ideal em comunidades diversas são fundamentais para o sucesso na gestão da SCRM. Essas iniciativas não apenas contribuem para a melhoria dos desfechos clínicos, mas também fomentam uma abordagem mais equitativa e sustentável para a saúde de todos os pacientes afetados.

## Conclusão e Mensagem Final

A SCRM oferece uma nova perspectiva sobre a inter-relação entre doenças cardiovasculares, renais e metabólicas. Integrar abordagens terapêuticas para essas condições, que compartilham mecanismos fisiopatológicos comuns, como resistência à insulina, inflamação crônica e disfunção endotelial, é fundamental para aprimorar o manejo e os desfechos clínicos dos pacientes. A adoção de estratégias de prevenção, triagem precoce e tratamento integrado pode reduzir significativamente a morbidade e mortalidade associadas a essas doenças interligadas. Ademais, a colaboração interdisciplinar entre cardiologistas, nefrologistas, endocrinologistas e outros profissionais da saúde é crucial para o desenvolvimento de planos de tratamento personalizados e integrados, que levem em consideração as complexas interações entre os sistemas cardiovascular, renal e metabólico. Vale ressaltar que o novo conceito da SCRM “abraça” a importância da doença renal crônica. Enquanto o conceito clássico da síndrome metabólica representa um conjunto de alterações metabólicas que afetam o risco cardiovascular, a SCRM oferece uma nova visão, na qual alterações metabólicas decorrentes da obesidade disfuncional estão ligadas a alterações cardíacas e renais, configurando um ciclo de disfunção que demanda uma abordagem global. No contexto atual, em que o mundo vive uma pandemia de obesidade, DM2, hipertensão e DRC, a SCRM emerge como um conceito atual e de extrema importância.
